# Gustatory receptor 11 is involved in detecting the oviposition water of Asian tiger mosquito, *Aedes albopictus*

**DOI:** 10.1186/s13071-024-06452-w

**Published:** 2024-08-29

**Authors:** Si Yu Zhao, Pei Lin Wu, Jun Yu Fu, Yi Ming Wu, Hong Kai Liu, Li Jun Cai, Jin Bao Gu, Xiao Hong Zhou, Xiao-Guang Chen

**Affiliations:** Department of Pathogen Biology, Institute of Tropical Medicine, School of Public Health, Southern Medical University, Guangzhou, China

**Keywords:** *Aedes albopictus*, Oviposition, Small water container, Gustatory

## Abstract

**Background:**

*Aedes albopictus* is a major arbovirus vector with small stagnant water containers being its oviposition sites. Mosquitoes search for these sites based on their olfactory cues (odor and moisture emanating from the water at the oviposition site), visual cues (size and color of the site), and gustatory cues (ion and nutrient concentration in that water). The gustatory mechanism through which mosquitoes search for oviposition sites remains unknown.

**Methods:**

To investigate the role of taste receptors in *Ae. albopictus* oviposition site selection, we developed a laboratory model. This model assessed mosquito behavior in locating and detecting oviposition sites, using a location index to quantify site preference and detection time to measure response to water presence. We compared oviposition site-searching efficiency between mosquitoes with blocked and unblocked appendages, targeting the taste organs. Transcriptome sequencing was conducted to identify differentially expressed genes between water-exposed and unexposed mosquitoes. CRISPR/Cas9 technology was then employed to generate a mutant strain with a targeted gene knockout.

**Results:**

There was no significant difference between the blocked and unblocked groups in the location index. In contrast, the detection time of the unblocked group differed significantly from all other groups, including those with blocked foreleg tarsus, midleg tarsus, hindleg tarsus, all tibia, and all tarsus. Transcriptome sequencing analyses of water-exposed and unexposed mosquitoes revealed that the taste-related gene gustatory receptor 11(*gr11*) was differentially expressed. This gene was knocked out with CRISPR/Cas9 technology to generate a pure mutant strain with 2- and 4-bp deletions, which exhibited a significantly longer detection time than the wild-type strain.

**Conclusions:**

This study reveals the role of *Ae. albopictus gr11* in water detection at oviposition sites, thereby providing a theoretical basis and scientific guidelines for managing the breeding sites of these mosquitoes.

**Graphical Abstract:**

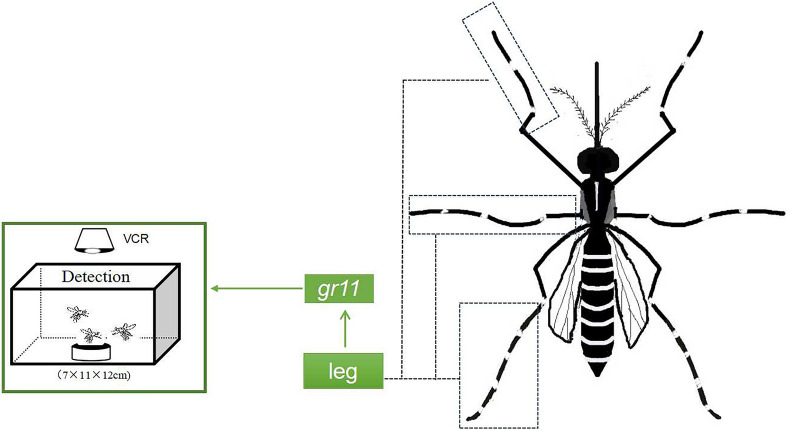

**Supplementary Information:**

The online version contains supplementary material available at 10.1186/s13071-024-06452-w.

## Background

*Aedes albopictus* is a crucial arbovirus vector and can transmit dengue virus, Zika and chikungunya virus, and others. *Aedes albopictus* is considered one of the most invasive species globally. It has spread from Asia, its original habitat, to worldwide, except Antarctica, in fewer than 50 years [1–3]. Three-quarters of a mosquito's life, including the growth and development of eggs, larvae, and pupae, is completed in the water at the oviposition site [4], with the pupae only flying away when they turns into adult mosquitoes. Therefore, oviposition sites are critical for mosquito control. Various sensory organs of mosquitoes play a vital role in their oviposition site search [5, 6].

The oviposition waters typically include plant leachates, metabolites of other or similar species, and microbes and their metabolites [4, 7–9]. Mosquitoes perceive these oviposition sites through various olfactory and gustatory cues. Some of the gustatory cues are perceived not through mouthparts but as appendage-dominated gustatory contacts [10]. Mosquito legs probing the water during oviposition have been reported [11]. Furthermore, chemosensory organs on the insect foot play a crucial role in taste perception [12].

Gravid mosquitoes come in contact with the water where they lay their eggs to assess its suitability for egg laying, including evaluation of chemical signals such as salinity, and presence of acidic or sweet compounds [13]. The ppk301 channel of *Ae. aegypti* mediates the perception of water and their salt concentrations [11], with some specific mechanisms involving osmotic pressure and sodium ion recognition. PPK genes, members of the protein/ Epithelial sodium channel (ENaC channel) superfamily, encode cation channel subunits. These subunits act as mechanoreceptors and chemoreceptors that can be stimulated by various stimuli including pheromones, fluid osmolality, and salt [14].The GR (gustatory receptor) family of genes, first identified in *Drosophila* [15], is predominantly found in gustatory organs. They play critical roles in various physiological behaviors including feeding, toxin avoidance [16], courtship, mating, and egg laying [17]. *Gr21a* and *Gr63a* are CO_2_ receptors in *Drosophila* [18].

In mosquitoes, searching for a suitable oviposition sites is a complex process combining olfactory, visual, and gustatory stimuli. Different mosquito species exhibit varying preferences regarding oviposition sites. For instance, *Culex pipiens pallens* and *C. quinquefasciatus* favor breeding in stagnant water, particularly where the water is visibly dirty and contains a high organic matter concentration [19]. Conversely, *Ae. albopictus* frequently breeds in small, clean stagnant water, such as that found in empty cans, tires, bamboo, and wood holes [20]. Determining the involvement of mosquito legs in searching for oviposition and identifying the underlying molecular mechanisms would be advantageous. To address this question, this study first established a model of *Ae. albopictus* searching for oviposition sites. This was then examined by blocking the mosquito legs, followed by the screening of the effect of this blocking on the search for oviposition sites through a comparative analysis of transcriptomes of mosquitoes from water in contact with and not in contact with oviposition sites. Finally, CRISPR/Cas9 technology was used to knock down the differential expressed genes, and the effect of this knockdown on the search for the oviposition sites was observed.

## Methods

### Mosquito strains and breeding

The Foshan strain of *Ae. albopictus*, a gift from the Guangdong Center for Disease Control and Prevention, was routinely reared in the laboratory at a temperature of 27 °C ± 1 °C, relative humidity of 65% ± 10% under a 14-h light/ 10-h darkness cycle.

### Blocking methods

Drawing on the previous methods used in *Drosophila* [21] and *Ae. aegypti* [10], glue (Kraft Shadowless Glue, K-303) was applied to the tibial and tarsal segments of mosquito legs by using a brush (0000# Hook and Loop, Xie De Tang, Beijing). The glue was dried through illumination with a UV lamp for 30 s. The treatment groups were categorized into foreleg tarsal segments, midleg tarsal segments, hindleg tarsal segments, all tibial segments, and all tarsal segments. The control group was not treated with any ointment and was only exposed to UV lamp for 30 s.

### Oviposition site section assay

Three-hole ovitraps are generally used as trapping containers during mosquito breeding site monitoring [22]. Inside a 1-m^3^ mosquito net, three-hole ovitraps were placed diagonally. These traps consisted of a clear cylindrical plastic jar (height: ~ 10 cm, diameter: ~ 5.5 cm at the bottom) with a recessed bottom and a black lid with three tapered small holes of the same size (top diameter: 7 mm, bottom diameter: 5 mm). This design was used to reduce the number of escaping mosquitoes (Additional file 1A). One jar contained 100 ml dechlorinated water, and the other jar was left blank. Three days after blood sucking, 20 gravid mosquitoes were placed in the mosquito nets. The number of gravid mosquitoes in the three-hole ovitraps was recorded after 6 h. Location index = number of mosquitoes that searched the three-hole ovitraps with water/total number of mosquitoes released. The location index of both the leg-appendage-blocked mosquitoes and the normal-leg mosquitoes was measured using the aforementioned formula. Each experimental group was repeated six times. The temperature and humidity were set at 28–30 °C and 40–50%, respectively. The location index indicates the efficiency of the mosquito's sensory organs to sense the characteristics of the oviposition site. The location index ranges from 0 to 1. The higher the location index is, the better the mosquito's oviposition site-searching ability. These mosquitoes mainly feed on the blood of Kunming mice.

### Modeling mosquito detection of oviposition sites

The time at which mosquitoes rested on the inner wall of the container or touched the water surface was considered to examine the start of egg laying, while observing them with abdominal curling and leg detection until the appearance of white eggs on the surrounding water surface. The time from their contact with the water to the laying of the first egg was the detection time (measured in seconds). The complete process was observed on video (Logitech, model Streamcam, 1080 p, 60 fps).

Experimental procedure: First, a video camera was placed above a 7 × 11 × 12 cm^3^ customized mosquito cage (Additional file 1B). Three days after blood feeding, 10 gravid mosquitoes were invested. A small water cup holding 30 ml water was placed inside the mosquito cage, with a piece of black cloth embedded in the cup to observe white mosquito eggs. Video recordings were started from 3:00 p.m. to 6:00 p.m. The video recorded 2–3 replicates for the same treatment factor. Each video has multiple mosquito detections of the water recorded. Detection time was used to measure the response of mosquitoes in close proximity to water, including smell, sight, and taste. The shorter the time, the more suitable the water is for egg laying, as perceived by the mosquito.

### Detection of mosquito leg transcriptome analysis at oviposition sites

Test group: After 3 days of blood feeding, gravid mosquitoes were released in the mosquito cage. The mosquito cage was placed in a container containing deionized water. The activities of the mosquitoes were continuously observed. The mosquitoes with their legs contacting the water surface and those exhibiting abdominal curving were immediately treated with liquid nitrogen. Mosquitoes laying eggs were not considered. In total, leg tissue was obtained from 40 treated mosquitoes, each with six complete legs. This group was recorded as the test leg group exhibiting contact with the water surface.

Control group: For the experiment, gravid female mosquitoes were allowed to feed on blood for 3 days before being released into a mosquito cage. However, the cage was then placed in a container that did not contain dechlorinated water. Synchronized collection of 40 mosquito legs was performed. This group was recorded as the control leg group without water contact. The experiment was repeated three times.

The collected mosquito leg tissue was fully ground and preserved in Trizol. The tissue samples were sent to Beijing Novozymes Sequencing Company for transcriptome sequencing. Illumina sequencing generated 150-bp paired-end reads after raw data filtering and pooling of different libraries, following requirements for effective concentration and target off-machine data volume.

From the obtained raw sequencing data (raw reads), low-quality reads containing splice adapters, reads with unidentifiable base information accounting for > 10% of the entire sequence, and reads with a sequencing error rate Qphred of ≤ 20 bases accounting for > 50% of the entire length were filtered to gain clean reads for subsequent bioinformatics analysis.

Refer to the following genomes: https://ftp.ncbi.nlm.nih.gov/genomes/all/GCF/006/496/715/GCF_006496715.2_Aalbo_primary.1/ (GCA_006496715.1).

We defined genes with a fold change (FC) of more than onefold (|log2FoldChange|≥ 0) and a *P* ≤ 0.05 as significantly differentially expressed genes. Based on the DEGs obtained, chemosensory-related genes were identified according to each gene description and compared using the Blastn tool in NCBI to reconfirm the accuracy of gene sequences and nomenclature. Information such as the number of DEGs and reads is presented in Additional files 4 and 5.

### Reverse transcription-quantitative real-time PCR detection of differentially expressed genes(DEGs)

The samples were validated for RT-qPCR in the same manner as the sequencing samples. The collected tissues were well ground, and tissue RNA was extracted using the Trizol reagent (Ambion, Life Technologies, Carlsbad, CA, USA). Tissue RNA was diluted and dissolved in 1.5-ml of enzyme-free EP tubes by using 20 μl RNA Nase-free water. A trace amount of RNA was collected to determine its quality by using Nanodrop 2000. The cDNA strand was synthesized by removing the genome and subjecting it to reverse transcription by using oligo-(dT) primers and the Reverse Transcription Kit (Promega Corporation, Madison WI, USA). Genomic DNA was removed using the TURBO DNA-free Kit (Ambion, Life Technologies, USA). After cDNA had been obtained, reverse transcription-quantitative real-time PCR (RT-qPCR) was performed. The PCR program was as follows: heating at 95 ℃ for 10 s, 60 ℃ for 15 s, and 72 ℃ for 20 s for a total of 40 cycles. Gustatory receptor 11 (LOC 109412825) mRNA expression was compared with the relative mRNA expression of the internal reference gene (actin-5C, LOC109405344) by using the 2^−ΔΔCt^ method. Additional file 4 provides primer information.

### Cas9/guide RNA editing experiments for *gr11* genes

Gene editing was performed referring to previous methods [23–25]. First, the online website (http://crispr.mit.edu) was employed to design a suitable guide RNA. Then, Q5 high-fidelity DNA polymerase was used to anneal and ligate the double-stranded primer (sgRNA-F/R) to generate the in vitro transcription template of sgRNA. The PCR products were subsequently obtained, and DNA Fragment purification Kit version 4.0 was used for purification. The DNA fragment concentration was determined using the NanoDrop2000 Nucleic Acid Concentration Meter. Using the T7 Transcription Kit T7RiboMAX^™^ Express Large Scale RNA Production System, the purified DNA fragments were employed as templates for in vitro transcription. The injection mixes contained 300 ng/ul Cas9 Protein (Thermos Fisher Scientific), 100 ng/ul purified sgRNA1, and 100 ng/ul purified sgRNA2 added to RNase-free water. The injected mosquito eggs were placed in a climatic chamber and incubated for 48–72 h. The larvae were transferred to a new container and replenished with dechlorinated water. The larvae were maintained until they reached the adult stage. At this point, the adult mosquitoes were reared in a climatic chamber and provided with 10% glucose solution. During rearing, data on the hatching, pupation, and eclosion rates were recorded. Genomic DNA extracted from the mosquito legs by using the Extract-N-Amp Tissue PCR Kit was screened for mutant G_0_ individuals. The mutant G_0_ adult mosquitoes were mated with wild-type female or male mosquitoes to further screen for G_1_ and obtain individuals with heritable mutation types. We screened the G_0_ generation of mutant mosquitoes using PCR. For the G_1_ generation, we employed a combined approach of PCR and sanger sequencing. These individuals were then self-crossed to achieve a purely synonymous mutant line [23]. Once pure mutant lines were obtained, the *gr11* conserved and transmembrane structural domains were predicted and their amino acid structures were mapped using the online websites https://www.ncbi.nlm.nih.gov/Structure/cdd/wrpsb.cgi and https://dtu.biolib.com/DeepTMHMM.

### Relative expression assay of *gr11* gene transcript levels

To verify *gr11* gene transcript levels in the pure mutant strain, samples were collected from the key stages of mosquito growth and development. The samples were collected as 10 larvae for one pool, 5 pupae for one pool, 5 female mosquitoes for one pool, and 5 male mosquitoes for one pool. To exclude other interfering factors, the wild-type group was launched at the same time as the mutant strain group. The tissue (larvae, pupae, female mosquitoes, male mosquitoes) was ground using a grinder, and tissue RNA was extracted using the Trizol method. After RNA of suitable concentration and quality was obtained, cDNA was synthesized through reverse transcription. RT-qPCR was also performed. Primer information is presented in Additional file 3.

#### Larval development and female mosquito fertility experiment

The egg paper was incubated in water. A group of 50 first-instar larvae were then reared in a 300-ml plastic bowl until all larvae reached the pupal stage. Pupae were individually isolated in tubes for synchronous observation of emergence.

The number of surviving larvae and pupae and mosquitoes that fledged were recorded until all larvae fully developed into adult mosquitoes. In total, three biological replicates were maintained under the following rearing conditions: temperature of 27 °C ± 1 °C, humidity of 70–80%, and 16 h light/8 h dark cycles.

We put each gravid mosquito, 3 days after blood feeding, into a 300-ml cup with a humid environment. After 24 h, we counted the eggs laid by wild-type and gr11 mutant mosquitoes with a total of 24 repetitions.

### Statistical analysis

Statistical analysis was performed using SPSS version.21 (IBM SPSS Statistics). All data were analyzed using the Shapiro-Wilk normality test (*α* = 0.05). The following parameters were tested according to the condition of normal test. The location index was analyzed using a chi-squared test.

The location index, larval pupation rate, and eclosion plumage rate were analyzed using a chi-square test. The detection time and the number of eggs laid were analyzed using an independent samples t-test. *P* < 0.05 was considered to indicate a statistically significant difference.

## Results

### Tests for searching oviposition sites for blocked *Ae. albopictus* legs

**Fig. 1:**
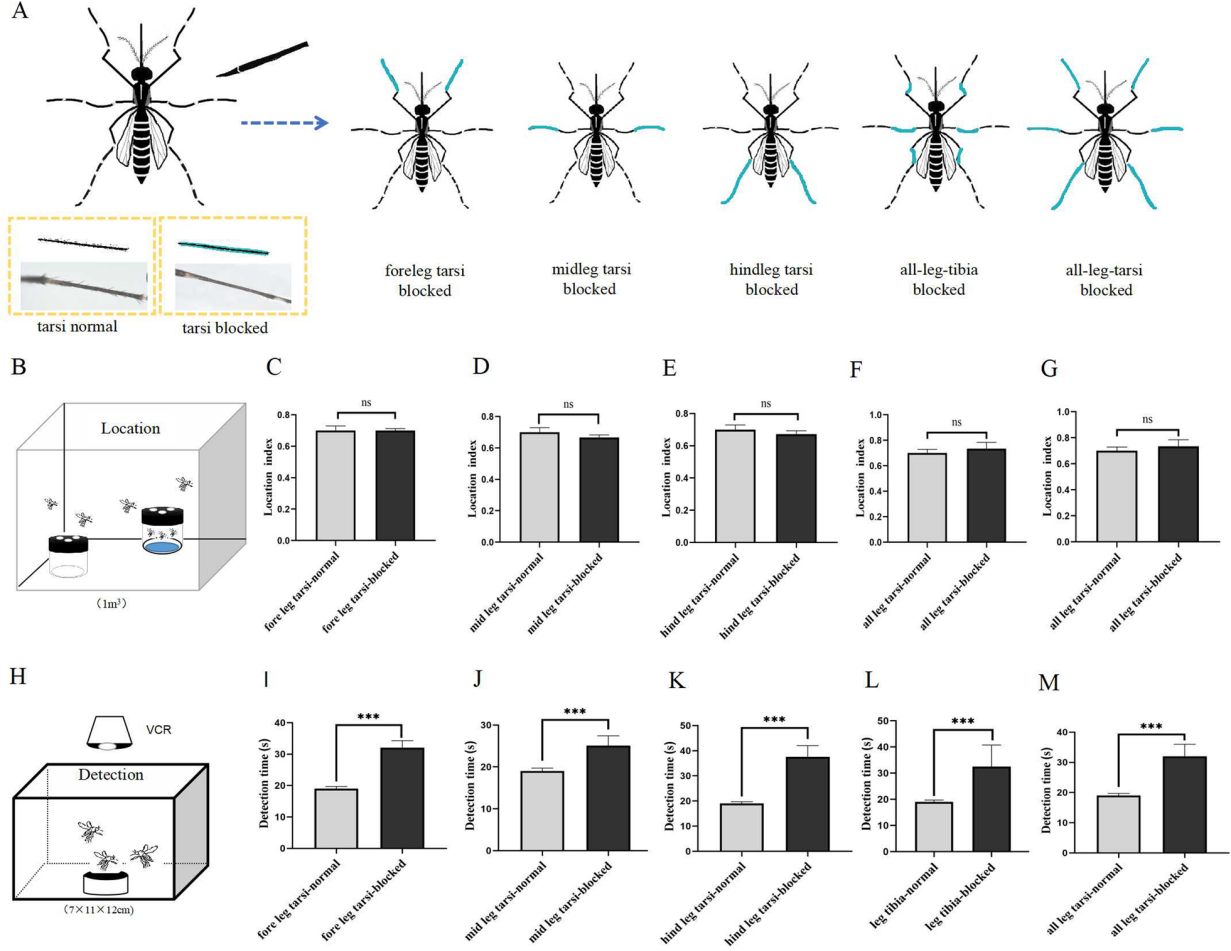
Comparison of location index and detection time between mosquito leg-blocked and unblocked groups. **A** Patterns of different parts of blocked mosquito legs. Tarsi normal represents normal tarsi without blocking, whereas tarsi blocked represents the experimental group with blocking closure. Foreleg tarsi blocked represents foreleg tarsi closure, midleg tarsi blocked represents midleg tarsi closure, hindleg tarsi blocked represents hindleg tarsal closure, all-leg-tibia blocked denotes all tibial closure, and all-leg-tarsi blocked represents all tarsal closure. **B** A diagram of the location stage pattern with a three-hole ovitrap containing 100 ml water and a waterless three-hole ovitrap placed diagonally. **C** A comparison of location index between the blocked foreleg tarsal limbs and the unblocked group (*P* > 0.05, *N* = 6). **D** A comparison of location index between the blocked middle leg tarsal limb and the unblocked group (*P* > 0.05, *N* = 6). **E** A comparison of location index between the blocked hind leg tarsal limb and the unblocked group (*P* > 0.05, *N* = 6). **F** A comparison of location index between all blocked tibial segments and the unblocked group (*P* > 0.05, *N* = 6). **G** A comparison of location index between all blocked tarsal segments and the unblocked group (*P* > 0.05, *N* = 6). **H** Pattern map of detection stages. **I** A comparison of mosquito detection time between the blocked forelimb tarsus and unblocked group (*t* =  − 7.413, *df* = 415, *P* < 0.001). **J** A comparison of detection time between the unblocked group and closed mid leg tarsus (*t* =  − 3.22, *df* = 415, *P* < 0.001). **K** A comparison of detection time between the unblocked group and closed hindlimb tarsal limb (*t* =  − 6.407, *df* = 351, *P* < 0.001). **L** A comparison of tibial limb detection time in the unblocked group and closure of all tibial limbs (*t* =  − 3.741, *df* = 320, *P* < 0.001). **M** A comparison of detection time in the unblocked group versus closure of all tarsal limbs (*t* =  − 5.446, *df* = 362, *P* < 0.001). All data in the graphs were analyzed through t-test. Error lines represent standard errors, *P < 0.05, **P < 0.01, ***P < 0.001, NS, *P* > 0.05

The process *Ae. albopictus* searching for oviposition sites was divided into the stages of locating containers (location) and detecting water (detection). The location index refers to the proportion of released mosquitoes that searched for small stagnant water containers for gravid mosquitoes. A higher location index indicates a stronger ability of the mosquitoes to locate oviposition sites. The detection stage is based on the detection time, which is the time from which the mosquito comes in contact with the water surface to the time it lays the first eggs. A shorter detection time indicates that the water detected by the mosquito is more suitable for egg laying. According to the location stage results, the location index of blocked foreleg tarsus, midleg tarsus, hindleg tarsus, all tibial segments, and all tarsal segments were not different from those of the unblocked group (Fig. 1C–G, *P* > 0.05). This indicated that the mosquito leg tissues were not involved in localizing the egg-laying containers. When detecting the water, the detection time of the unblocked group, foreleg tarsus-blocked group, midleg tarsus-blocked group, hindleg tarsus-blocked group, all tibia blocked-group, and all tarsus-blocked group were 19.01 ± 0.7, 32.09 ± 2.18, 25.10 ± 2.30, 28.85 ± 4.21, 32.50 ± 8.28, and 31.98 ± 4.02 s, respectively. The differences in detection time between and the blocked and unblocked mosquitoes were statistically significant (Fig. 1I–M. *P* < 0.001). The results thus suggested that both tibial and tarsal segments of the mosquito legs were involved in water detection.

### CRISPR/Cas9 editing of the *gr11* gene

**Fig. 2:**
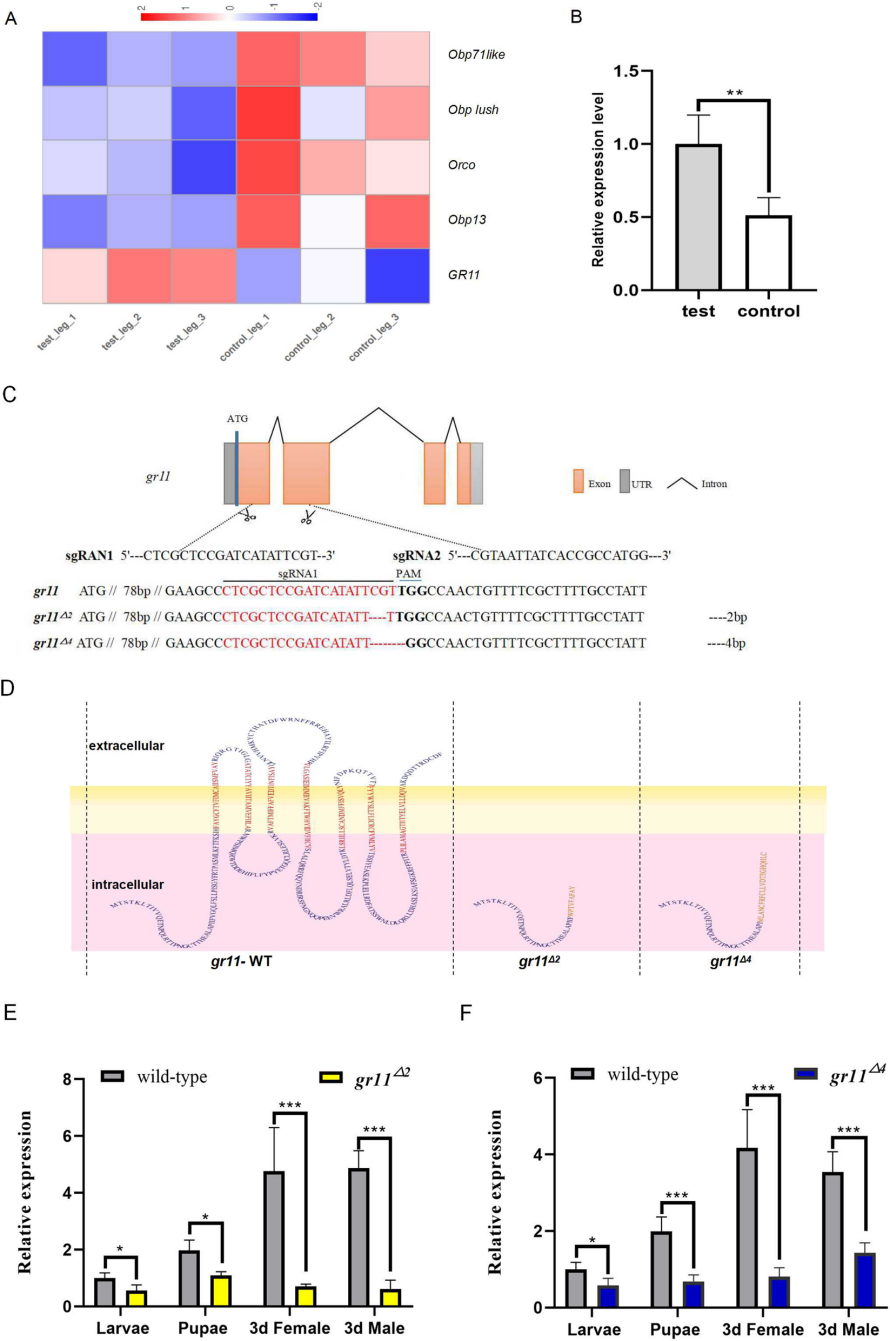
Transcriptome and RT-qPCR analyses of mosquito legs exposed and unexposed to water. **A** A heatmap of differential genes associated with chemosensitivity in the transcriptome of mosquito legs that came in contact with water and their counterpart. Detailed data are presented in Additional file 4. The experimental groups were noted as test-leg-1, 2, and 3. The control groups were noted as control-leg-1, 2, and 3. **B** Tissues of mosquito legs that came in contact with water and their counterpart. RT-qPCR analysis of the *gr11* gene. **C** A schematic of *gr11* knockdown and mutation types. The red characters are sgRNA and the bold black characters are PAM. The short-term line represents the missing bases; 2 and 4 bases were missing, respectively. **D** Structural prediction of wild-type *gr11*, *gr11*^*∆2*^, and *gr11*^*∆4*^ proteins. The leftmost part indicates the amino acid structure of the wild-type *gr11*, and the middle and rightmost parts indicate the predicted transmembrane regions of the *gr11*^*∆2*^ and *gr11*^*∆4*^ proteins, respectively. Intracellular stands for intracellular, extracellular stands for extracellular, and the yellow area represents the public transmembrane schematic. **E** A comparison of mRNA expression between the wild-type and *gr11*^*∆4*^ strains in growth and developmental stages, with samples obtained from male and female mosquitoes that were not mated at larval, pupal, and 3 days post-eclosion. **F** A comparison of mRNA expression between the wild-type and *gr11*^*∆2*^ strains at growth and developmental stages, with samples obtained from male and female mosquitoes that were not mated at larval, pupal, and 3 days post-eclosion. All data presented in the graphs were analyzed using a t-test. Error lines represent standard errors, *P < 0.05, **P < 0.01, ***P < 0.001, NS, *P* > 0.05

Based on the results of searching for oviposition sites by using *Ae. albopictus* appendages, the functional molecules involved were explored. The legs of the 3 days after blood feeding mosquitoes, which were in contact with the water surface but did not lay eggs, were used as the experimental group. By contrast, the legs of the 3-day after blood feeding mosquitoes, which were not in contact with the water surface, were collected as the control group. We also screened five differentially expressed chemosensory-related genes: odorant-binding protein 71like (LOC109428879), odorant-binding protein lush (LOC109401200), odorant receptor co-receptor (*orco*) (LOC109399318), odorant-binding protein 13 (LOC109416735), and gustatory receptor 11 (LOC109408683). Of them, the expression of only *gr11* was upregulated (Additional file 4; Fig. 2A).

RT-qPCR analysis of the *gr11* gene was performed to verify the accuracy of this sequencing result. Leg tissues of female mosquitoes exposed to water were used as the experimental group and those from female mosquito not exposed to water were used as the control group. The experimental group exhibited higher *gr11* gene expression than the control group, and the difference was statistically significant (Fig. 2B, *P* < 0.01).

When in close contact with the water surface, mosquitoes use their legs to explore the suitability of the water as an oviposition site. Chemosensory functions are noted in the tarsal segments of the legs of *Ae. aegypti* mosquitoes [10]. *Drosophila* tarsus was associated with exposure to octanoic acid, which is preferred for the oviposition [26]. The comparative transcriptome analysis of the mosquito leg-contacted water surface group and uncontacted water surface group unveiled that *gr11* gene was the only upregulated gene associated with chemosensory receptors. We hypothesized that the *gr11* gene might be involved in water detection at the oviposition site by *Aedes albopictus* gravid mosquitoes. The *gr11* gene was therefore knocked out through CRISPR/CAS9 gene editing, and the gene function was analyzed. The *gr11* gene has four exons and three introns (Fig. 2C), and double sgRNAs are located on the first and second exons. The structural domains of the gene were predicted by referring to a website with seven transmembrane structural domains (Fig. 2B).

The sgRNA and Cas9 enzyme mixture was microinjected into the eggs at 1 h post-laying, and *gr11* gene mutation in the G_0_ adult mosquitoes was detected through PCR. Ultimately, 6 mutant adult mosquitoes were obtained from 632 injected eggs (Additional file 3). Two mutation types of the pure heterozygotes were noted in the G_3_ adult mosquitoes (Fig. 2C) and were labeled as *gr11*^*∆2*^ and *gr11*^*∆4*^, respectively. They were a code-shifting mutation. At the protein level, both mutants lost their transmembrane regions (Fig. 2D). The relative expression of the *gr11* mutant lines was also verified at the mRNA level (Fig. 2E, F). *gr11* gene expression started at the larval stage and was the highest in the adult mosquito stage. *gr11*^*∆2*^ and *gr11*^*∆4*^ exhibited a similar trend of *gr11* gene expression, with the expression decreasing from the larval stage to the adult mosquito stage (Fig. 2E, F). This mutation still involves some expression because it is a code-shift mutation, and the mRNA of the gene is not completely unexpressed.

The aforementioned results indicate that heritable pure lines with *gr11* gene mutations were obtained, and the two mutation types, *gr11*^*∆2*^ and *gr11*^*∆4*^, were more similar. The gene mutation lines sometimes have more severe fitness cost. The fitness cost of the mutation lines was analyzed in this study. Pupation rate of wild-type, *gr11*^*∆2*^, and *gr11*^*∆4*^ were 0.82 ± 0.02, 0.79 ± 0.02, and 0.93 ± 0.04, respectively. Eclosion rates were 0.96 ± 0.07, 0.86 ± 0.04, and 0.91 ± 0.07, respectively. Statistical analysis showed no significant differences in either pupation or eclosion rate between the genotypes and the wild type (Additional file 2, *P* > 0.05). Our findings indicate that *gr11* gene mutations have no significant impact on pupation or eclosion rate.

We also compared egg production in wild-type mosquitoes to two *gr11* mutant lines (*gr11*^*∆2*^ and *gr11*^*∆4*^). Wild-type mosquitoes laid an average of 75.75 ± 4.45 eggs, while the mutants laid significantly fewer eggs (44.34 ± 5.41 for *gr11*^*∆4*^ and 43.47 ± 4.29 for *gr11*^*∆2*^) (Additional file 2, *P* < 0.05). This reduced fecundity suggests that *gr11* is involved in the perception of oviposition site water.

### Involvement of the gr11 gene in water detection by *Ae. albopictus* gravid mosquitoes

**Fig. 3:**
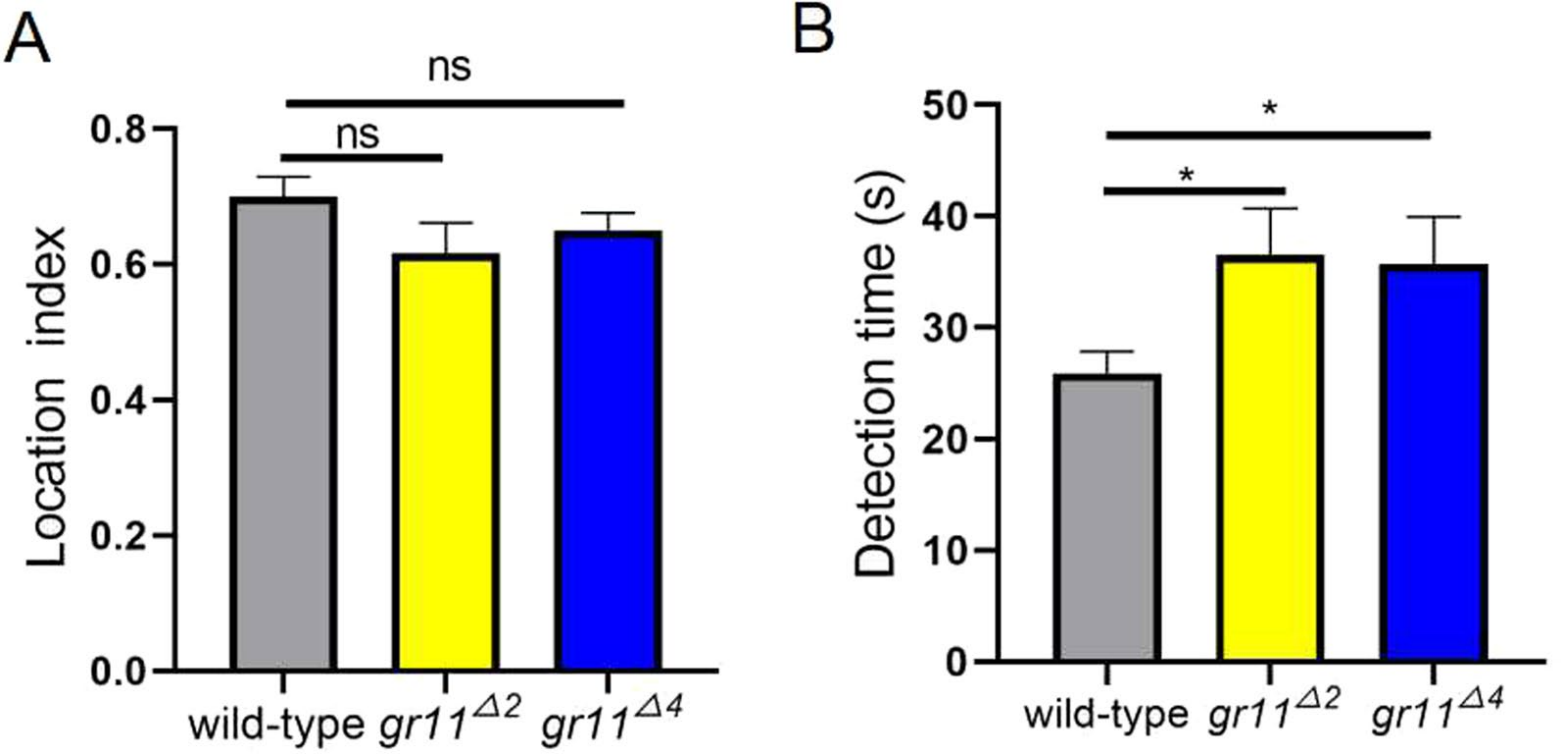
The *gr11* gene is involved in water detection by *Aedes albopictus* gravid mosquitoes. **A** A comparison of the localization index between the wild-type strain and both *gr11*^*∆2*^ and *gr11*^*∆4*^ strains (*P* > 0.05). **B** A comparison of detection time (in seconds) between the wild-type strain and both *gr11*^*∆2*^ (*t* = 1.581, *df* = 82, *P* < 0.05) and *gr11*.^*∆4*^ (*t* =  − 2.62, *df* = 113, *P* < 0.05) strains. All data in the graphs are expressed as mean ± standard error (means ± SEM). NS, *P* > 0.05, *P < 0.05, **P < 0.01, ***P < 0.001

The location indices were 0.70 ± 0.028 for the wild type, 0.61 ± 0.044 for *gr11*^*∆2*^, and 0.65 ± 0.025 for *gr11*^*∆4*^ (Fig. 3A, *P* > 0.05). The detection times were 25.88 ± 1.98, 35.69 ± 4.22, and 36.50 ± 4.18 s for the wild type, *gr11*^*∆4*^, and *gr11*^*∆2*^, respectively. The detection time of the mutants were significantly different from that of the wild type (Fig. 3B, *P* < 0.05).

Thus, the *gr11* gene was not involved in locating the oviposition sites but was involved in detecting them; *gr11* played a crucial role in the search of oviposition sites by *Ae. albopictus*.

## Discussion

We here demonstrated that appendages of *Ae. albopictus* play a crucial role in exploring water at oviposition sites. The differentially expressed *gr11* gene was screened through comparative transcriptome sequence analysis of mosquitoes exposed and unexposed to water. Furthermore, through *gr11* knockdown, we revealed the molecular mechanism underlying the exploration of water at oviposition sites.

Mosquitoes have elongated feet, and electron microscopy has shown that numerous different receptors are distributed on these feet [27]. Thus, they have a role in taste as they help recognize odors and detect small molecules in liquids [28, 29]. For example, the fifth tarsal segment of the forefoot of the female cotton bollworm has multiple hairy sensors responding to sucrose, glucose, fructose, maltose, lysine, and inositol [30]. *Gr* in *Drosophila* is involved in the detection of sugars and bitter substances [31]. *Gr5a, Gr43a, Gr64a*, and *Gr64f* genes are associated with sugar detection [32, 33]. *Gr33a*, *Gr66a*, and *Gr93a* are associated with responses that recognize caffeine and other bitter compounds [31, 34]. Additionally, *Ae. aegypti* mosquitoes use their legs to detect water for laying eggs [11]. These study results are consistent with the results and conclusions of the present study.

In addition to *gr11*, the DEGs included *obp71like* and *orco* genes, among others, which suggested that water detection also involves olfactory-related molecules. obp71like in *Ae. albopictus* is a direct homolog of the *Drosophila obp59a* counterpart. It was the first odor-binding protein reported to be associated with humidity perception [35]. *obp71like* is hypothesized to be involved in the detection of water. obp has diverse physiological functions [36]. Apart from being expressed in olfactory organs, obp is expressed in gustatory organs (including the rostrum and appendages). obp is hypothesized to be involved in recognizing gustatory substances, in addition to exercising olfactory functions [37]. For example, *Drosophila melanogaster obp57d* and *obp57e* are expressed in taste sensors present on the foot and contribute to the perception of pungent acids [26, 38]. Additional functional analyses of other differential genes screened need to be performed subsequently.

Mosquitoes use olfactory, visual, gustatory, and heat cues to select suitable oviposition sites, a complex behavior with multiorgan integration [39–42]. The *gr11* mutation does not completely disable the mosquito's ability to detect water and lay eggs. This suggests that mutant mosquitoes can rely on other cues for searching these oviposition sites. Olfactory cues include plant infusions, odors emanating from microbes, and presence of predators [9, 42–44]. Visual cues include container color and size [45–47]. Taste is used to determine whether the water has the appropriate osmotic pressure and whether it is sufficiently salty for offspring survival, such as the mechanosensory channel represented by ppk. *ppk301* is associated with freshwater oviposition in *Ae. aegypti* mosquitoes [11]*.*

Research progress in terms of visual, olfactory, and gustatory senses of mosquitoes searching for oviposition sites can aid is developing breeding site control technologies and products. For example, based on the preference of *Ae. aegypti* mosquitoes for black oviposition containers, black oviposition traps were developed in this study. When mosquitoes search for water in breeding sites, similar larvae present in the water may repel or attract gravid mosquitoes. This has led to the development of ovitraps and deterrents [48] and various olfactory attractants [49, 50]. By further elucidating the perceptual mechanisms of *Ae. albopictus* searching for oviposition sites, scientific guidelines for developing ovitraps for *Ae. Albopictus* can be drafted.

## Conclusions

This study reveals for the first time the role of the *gr11* gene present in the *Ae. albopictus* appendages in detecting small water and provides scientific guidelines for managing the breeding sites of these mosquitoes.

### Supplementary Information

Additional file 1. Experimental setup for oviposition site searching.

Additional file 2. Analysis of larval development and fecundity in mutant and wild-type strains.

Additional file 3: Table 1. Statistics of mutation rate of G_0_ adults in *Aedes albopictus.* Table 2. The primers used in this study. Table 3. The raw data for the location index in Fig. 3.

Additional file 4. Summary of all differentially expressed genes (Xcel file).

Additional file 5. Volcano plot illustrating the distribution of differentially expressed genes (DEGs) between the experimental and control groups.

## Data Availability

No datasets were generated or analysed during the current study.
