# Supplementary material for: Gustatory receptor 11 is involved in detecting the oviposition water of Asian tiger mosquito, Aedes albopictus
Source: Parasit Vectors. 2024 Aug 29;17:367. doi: 10.1186/s13071-024-06452-w (PMC11363565; doi:10.1186/s13071-024-06452-w)
Supplement: Supplementary file 1 — Additional file 1. Experimental setup for oviposition site searching. [file 13071_2024_6452_MOESM1_ESM.docx]

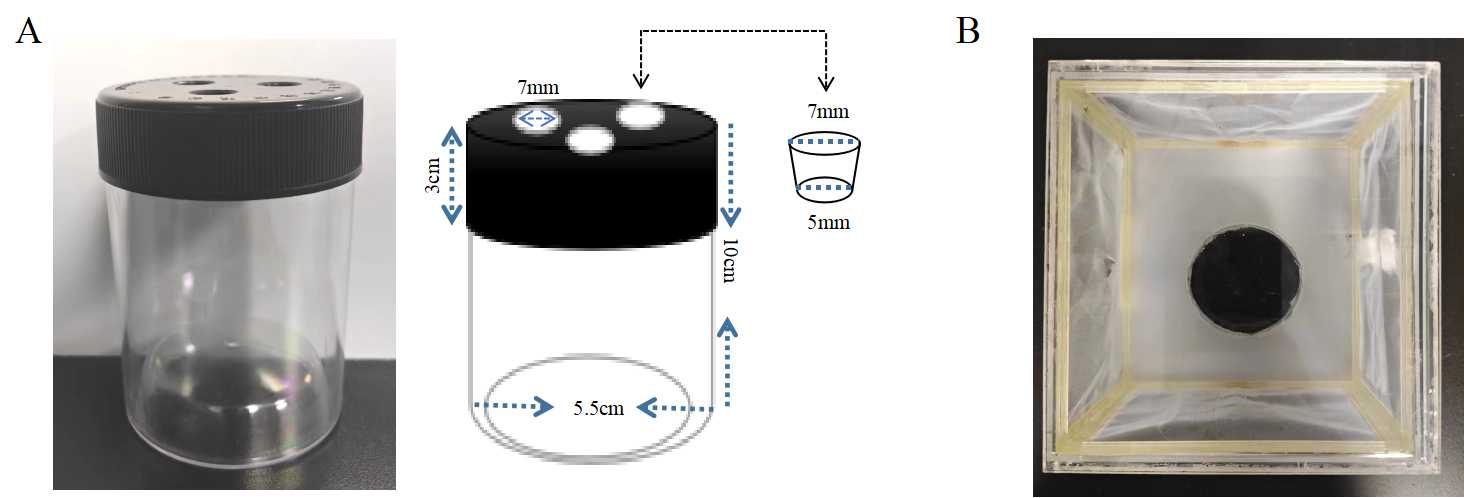


Additional file 1. Experimental setup for **oviposition** site searching. (A) Physical drawing and pattern diagram of a three-hole ovitrap with small holes in the black lid of a wide top and narrow bottom design (top diameter: 7 mm, bottom diameter: 5 mm). (B) The size of the container used for mosquitoes searching for oviposition site shooting was 7 × 11 × 12 cm^3^. The center container is inlaid with a piece of black cloth, so that the white mosquito eggs can be easily observed.
