# Supplementary material for: Gustatory receptor 11 is involved in detecting the oviposition water of Asian tiger mosquito, Aedes albopictus
Source: Parasit Vectors. 2024 Aug 29;17:367. doi: 10.1186/s13071-024-06452-w (PMC11363565; doi:10.1186/s13071-024-06452-w)
Supplement: Supplementary file 2 — Additional file 2. Analysis of larval development and fecundity in mutant and wild-type strains. [file 13071_2024_6452_MOESM2_ESM.docx]

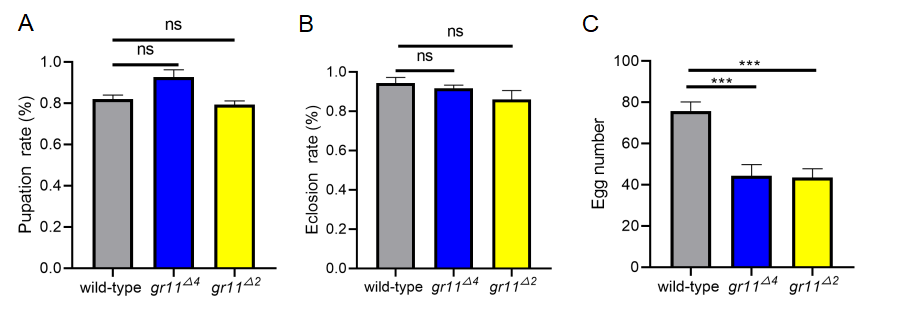


Additional file 2. **Analysis of larval development and fecundity in mutant and wild-type strains.**

(A) A comparison of pupation rates between the wild type and the *gr11* mutants (*gr11^∆2^*and *gr11^∆4^*) revealed no significant differences (*P* > 0.05, N = 3).

(B) A comparison of eclosion rates between the wild type and the *gr11* mutants (*gr11^∆2^*and *gr11^∆4^*) revealed no significant differences (*P* > 0.05, N = 3).

(C) Egg production of wild-type mosquitoes was significantly different from that of *gr11* mutants (*gr11^∆2^*and *gr11^∆4^*) (*P* < 0.001, N=24).All the data in the graphs are expressed as mean ± standard error (means ± SEM), NS, *P* > 0.05, **P* < 0.05, ***P* < 0.01, ****P* < 0.001.
