# Supplementary material for: Gustatory receptor 11 is involved in detecting the oviposition water of Asian tiger mosquito, Aedes albopictus
Source: Parasit Vectors. 2024 Aug 29;17:367. doi: 10.1186/s13071-024-06452-w (PMC11363565; doi:10.1186/s13071-024-06452-w)
Supplement: Supplementary file 5 — Additional file 5. Volcano plot illustrating the distribution of differentially expressed genes (DEGs) between the experimental and control groups. [file 13071_2024_6452_MOESM5_ESM.docx]

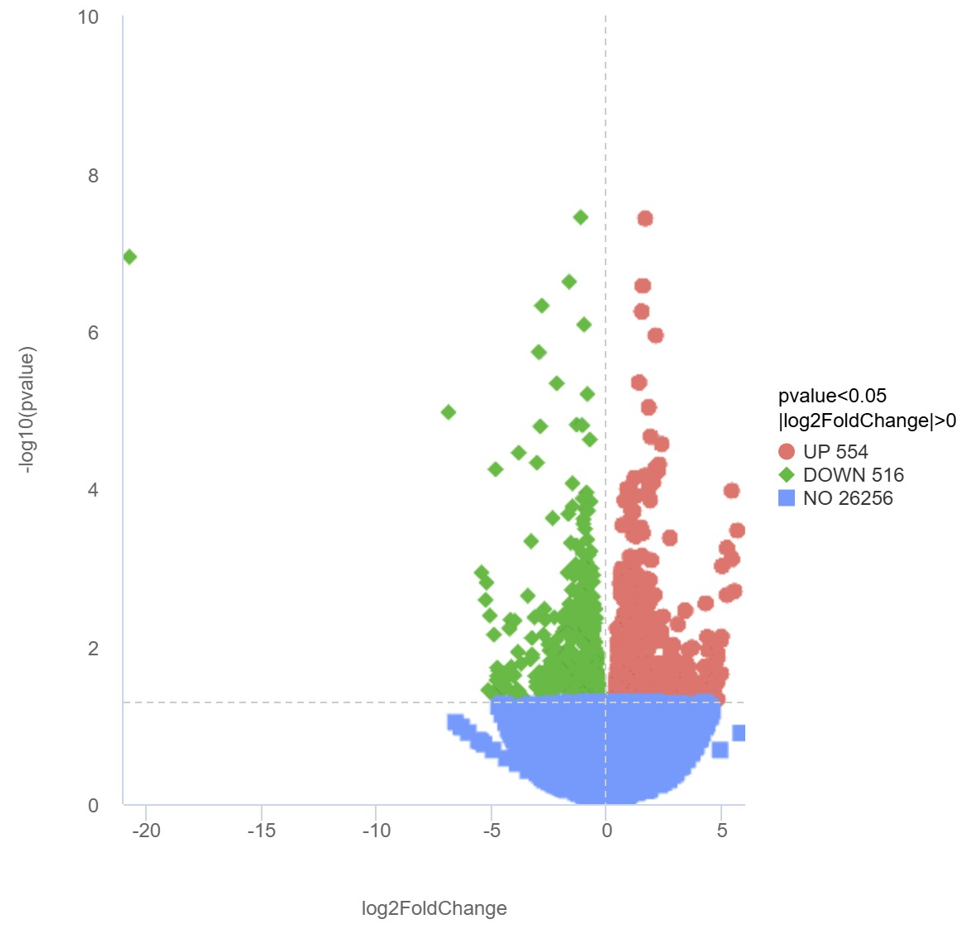


Additional file 5. Volcano plot illustrating the distribution of differentially expressed genes (DEGs) between the experimental and control groups. The x-axis represents log2FoldChange, and the y-axis represents -log10(p-value).
